# Linking Callous–Unemotional Traits to Social Withdrawal Among Young Chinese Left-Behind Children: Examining the Moderated Mediation Model

**DOI:** 10.3390/bs15030296

**Published:** 2025-03-03

**Authors:** Ruifeng Tan, Suiqing Chen, Xinying Guo, Huiyin Chen, Guixian He

**Affiliations:** 1 School of Education, Guangzhou University, Guangzhou 510006, China; tanrf@m.scnu.edu.cn (R.T.); gxy@e.gzhu.edu.cn (X.G.); 18316302058@163.com (G.H.); 2 School of Education, South China Normal University, Guangzhou 510631, China; 3 School of Journalism and Communication, Guangzhou University, Guangzhou 510006, China; riko2002@163.com; 4 Department of Education and Art, Luoding Secondary Vocational School, Yunfu 527200, China

**Keywords:** callous–unemotional traits, social withdrawal, emotion regulation, teacher–child relationship, left-behind children

## Abstract

Much attention has been given to the predictive role of callous–unemotional (CU) traits in children’s social and peer functioning. As an important dimension of social adaptation, early social withdrawal in preschool left-behind children (LBC) might be the outcome of psychological factors and growth environments. This study explored the predictive effect of CU traits on social withdrawal among preschool LBC, including the indirect effect of emotion regulation and teacher–child relationship quality. Data from 513 Chinese preschool LBC (257 boys and 256 girls) were reported by their teachers in rural areas, including assessments of CU traits, emotion regulation, social withdrawal, and teacher–child relationship quality. Path analyses showed that the CU traits of young LBC could significantly positively predict the prevalence of social withdrawal, and emotion regulation played a key mediating role in this effect path. Notably, teacher–child relationship quality moderated the indirect effect of emotion regulation on CU traits and withdrawal behaviors. Therefore, the moderated mediation model was supported. The findings expanded the recognition of LBC with CU traits and further suggested that the association of some personality traits and growing environments in the early left-behind childhood can significantly make a difference in peer functioning and social adjustment.

## 1. Introduction

With the development of rural revitalization since the reform and opening-up of China, the rural left-behind population has received increasing attention (Xu et al., 2023; Ma et al., 2022). Because parents migrate to cities, the positive development of behaviors and social engagement in their children left behind in rural hometowns has attracted serious attention (Wen & Lin, 2012). Preschool age (3–6 years old) is a vital developmental period for young children in terms of socialization and human capital accumulation (Knudsen et al., 2006; Jones et al., 2015). The formation of active cooperation and social interaction is of great significance to left-behind children (LBC).

However, the absence of parents for most of the year may result in insecure attachments in preschool LBC (Shuang et al., 2022). Furthermore, temporary guardians are committed to providing food and safe care, and the limited face-to-face interactions with migrant parents may increase the prevalence of emotional neglect and withdrawal behaviors (Ye & Lu, 2011; Zhan et al., 2021). The family transformation of LBC may make them actively lose their sense of presence in social interactions (Ye & Lu, 2011). A previous study identified an association between attachment and withdrawal responses (e.g., shyness and unsociability), especially avoidant attachment (Chen & Santo, 2016). Some researchers proposed that the construction of a developmental model consisting of biological, familial, sociodemographic, and extra-familial factors would contribute to the exploration of children’s active unsociability (Coplan & Weeks, 2010). Based on the general aggression model (GAM), aggressive behaviors might be the outcome of personal (e.g., personality traits) and situational factors (e.g., maladaptive families or parenting) through their effects on internal emotion variables (J. J. Allen et al., 2018; Anderson & Bushman, 2002). Additionally, from a comprehensive perspective through the GAM, withdrawn behavior might be a joint outcome influenced by the above influence factors (Shao & Zhang, 2021). For instance, an aggressive response may occur when there is an extreme aversion to developing and mending social relationships, while a withdrawal response may occur when there is a low desire to develop and maintain social relationships (Shao & Zhang, 2021).

Generally, social withdrawal is a precursor to social maladjustment in later childhood and adolescence (Ding et al., 2023). From an individual perspective, the increasing body of literature implies that CU traits might be regarded as a new predictor of low affiliative inputs and solitary activities in young children (Facci et al., 2023; Levine et al., 2023; Wagner et al., 2020; Hawes et al., 2019; Waller & Wagner, 2019; Porcelli et al., 2019; Haas et al., 2018). From an environmental perspective, LBC teachers have been recognized as unique figures providing attachment and emotional bonds (Liu et al., 2015; Commodari, 2013; Verschueren & Koomen, 2012). A positive relationship between LBC and teachers could weaken the risk of CU traits (Tan et al., 2023). Moreover, emotional expression and regulation may be closely associated with secondary CU traits from disadvantaged family experiences (Waller & Wagner, 2019; Lahey, 2014).

Existing studies have indicated that CU traits can strongly predict the early emergence and the extent of externalizing symptoms (Longman et al., 2016), but few studies have explored peer functioning and withdrawal symptoms (Facci et al., 2023). In particular, the effects of CU traits on social withdrawal among preschool LBC without parental care deserve further examination.

### 1.1. CU Traits and Social Withdrawal

CU traits generally refer to deficits in the subset of affection (e.g., empathy and guilt) and are precursors to serious externalizing syndrome in children and adolescents (Longman et al., 2016; Frick et al., 2014). However, children with CU traits might have difficulties developing and maintaining friendships (Miron et al., 2020) or even less desire to have and make friends (Haas et al., 2018). According to the sensitivity to threat and affiliative reward models, a low level of affiliative reward sensitivity or social motivation might be one of the inter-dimensions of CU traits (Waller & Wagner, 2019). From the psychopathological perspective of detachment (De Caluwé et al., 2019), there are relationships among anhedonia, intimacy avoidance, depression, restricted affectivity, and withdrawal (Holden et al., 2015). A previous study found that low-empathy children show greater social withdrawal and poor prosocial behaviors (Findlay et al., 2006).

Social withdrawal generally refers to a child’s behavior of removing themselves from interpersonal interactions (Rubin & Chronis-Tuscano, 2021; Rubin et al., 2009). Social withdrawal is detected in the first few years of life and is directly correlated with social and emotional development (Zhou et al., 2021; Guedeney et al., 2014; Milne et al., 2009). Different subtypes of social withdrawal behavior generally underlie children’s social motivation (e.g., approach and avoidance) (Coplan et al., 2013). For example, shy children tend to have higher avoidance motivation in social situations, whereas unsociable (i.e., socially disinterested) children tend to have lower approach motivation in interpersonal interactions. A prior study examined the impact of conflict, shyness, and unsociability on the prevalence of psychopathology during childhood (Kopala-Sibley & Klein, 2017).

Do young children with high CU traits care about social preference and friendships in real life? In fact, compared with their counterparts with low CU traits, individuals with high CU traits show a lack of sensitivity to self-performance and punishment in academic or athletic performance (Haas et al., 2015). However, little evidence implies that children and adolescents with high CU traits prefer solitude and fail to feel lonely (Haas et al., 2018). Children with higher CU traits may be mindful of whether peer functioning is valuable and decide whether to cooperate with others (Hawes et al., 2019). While a study on general adolescents found no significant link between CU traits and social withdrawal (Voss, 2022), this might not apply to possible behavioral outcomes in early childhood, especially in young children during critical periods of socialization. Furthermore, limited parental warmth and secure attachment were linked to CU traits in preschool-aged children (Baroncelli et al., 2024; Glenn, 2019; Waller et al., 2018). Therefore, it is reasonable to expect that CU traits would positively predict social withdrawal in LBC at the preschool age.

### 1.2. The Mediating Role of Emotion Regulation

Generally, emotion regulation involves attempts to recognize, control, modulate, and express emotional reactions in ways that support adaptive functioning (Gratz & Roemer, 2004; Cicchetti et al., 1995; Cassidy, 1994; Thompson, 1994; Garber & Dodge, 1991). An existing study identified the immediate and ongoing role of emotion regulation in cultivating a high degree of social competence (Blair et al., 2015). A higher ability to regulate emotions leads to a higher level of social interaction (Lopes et al., 2005). A previous study has found that emotional dysregulation could positively predict a decrease in prosocial tendencies in middle childhood (Elhusseini et al., 2023). In particular, the level of emotional self-regulation may affect social withdrawal during childhood (Calkins & Fox, 2002). Furthermore, affective experiences of shyness in social situations involve emotion regulation, particularly the down-regulation of negative emotions (Gross & John, 2002). A previous study showed that preschoolers who were socially hesitant and shy experienced emotional dysregulation (Rubin et al., 1995).

Are CU traits associated with emotion regulation? An existing study suggested a significant effect of emotion regulation on psychopathology involving emotional awareness, emotion regulation goals, and strategies (Gross & Jazaieri, 2014). A systematic review revealed that CU traits were robustly associated with emotion regulation (Squillaci & Benoit, 2021). A recent study indicated that preschool children with high CU traits have significantly poorer ratings of observed emotion regulation (Graziano et al., 2022). A prior study revealed that emotion regulation mediates the relationship between CU traits and antisocial behaviors (Falcón et al., 2021). Another study demonstrated the mediating role of emotion regulation in the association between personal disposition and withdrawal tendencies (Casini et al., 2022). Accordingly, regardless of the theoretical model (Shao & Zhang, 2021) or empirical findings (Kokkinos & Voulgaridou, 2022), emotion regulation may mediate the relationship between personality factors (e.g., CU traits) and withdrawal responses (e.g., social withdrawal).

Importantly, the prevalence of CU traits is closely associated with susceptible and maltreating parenting environments, where there might be a negative effect of CU traits on regulatory functioning as well (Waller & Wagner, 2019; Craig et al., 2023). Therefore, it is likely that emotion regulation mediates the relationship between CU traits and social withdrawal in young LBC.

### 1.3. The Moderating Role of Teacher–Child Relationship Quality

From the perspective of the relationship-driven model, teacher–child relationships affect the developmental pathway toward adjustment and maladjustment in young children (Zatto & Hoglund, 2019). A growing body of literature implies that high-quality teacher–child relationships contribute to developmental outcomes regarding social and cognitive skills in early childhood (Paes et al., 2023; Wu et al., 2018; Baker et al., 2008; Pianta & Stuhlman, 2004; Davis, 2003; Pianta et al., 1997). Therefore, children may perceive emotional support and trust from high-quality teacher–child relationships and learn to regulate and manage emotions in complex social situations to promote adaptive functioning (Berry & O’Connor, 2010; Pianta, 1999).

From the perspective of secure attachment accessible to LBC, teachers may provide emotional support and consideration for LBC and form multiple attachment relationships with these children (Liu et al., 2015; van Ijzendoorn et al., 1992). It was noted that the cultivation of emotion regulation was impacted by perceived environmental factors, such as attachments between children and adults (Maas et al., 2011). Drawing on the resilience framework (Kumpfer, 1999), as a protective factor, teacher–child relationship quality (TCRQ) contributes to children’s emotional resilience, further reducing the level of social maladjustment, such as the social withdrawal of LBC (Yang et al., 2021). Previous literature has posited that children’s emotion regulation is affected by components of emotional functioning in the family context (Morris et al., 2007). Despite this, high-quality teacher–child relationships provide prominent value and compensation for emotional adjustment in LBC with migrant parents (Liu et al., 2015; Xiang et al., 2022).

The existing studies indicate that disruptive behaviors are influenced by a combination of internal (e.g., CU traits) and external situational factors, which are mediated by internal emotional functioning (Tan et al., 2023; L. Zhang et al., 2023). The GAM posits that personal traits and situational variables also contribute to the prevalence of withdrawn behaviors by influencing inter-emotional function (Shao & Zhang, 2021). Prior studies have highlighted that high levels of teacher–student relationship quality promote students’ emotional prosperity rather than maladaptive outcomes such as CU traits caused by poor parenting practice (Baroncelli & Ciucci, 2020; Stoppelbein et al., 2021). The provision of high-quality teacher–child relationships is a significant developmental resource for young children (Hughes et al., 2012; Meehan et al., 2003). Moreover, the teacher–child relationship quality is of great significance to the psychological development of disadvantaged children (Y. F. Li, 2022).

In other words, the quality of the teacher–child relationship might play a protective role in the emotional development of LBC with limited parental care. There might be an interaction between CU traits and teacher–child relationship quality in predicting a child’s developmental outcomes (Crum et al., 2016). Therefore, it is implied that teacher–child relationships play a moderating role between CU traits and emotion regulation among young LBC during social interactions.

### 1.4. The Current Study

This study explored the relationship between CU traits and social withdrawal among left-behind young children. To understand the associations among variables, a hypothetical moderated mediation model was constructed and examined. To be specific, the following three hypotheses were tested:

**Hypothesis** **1.** *CU traits forecasts social withdrawal significantly.*

**Hypothesis** **2.** *Emotion regulation mediates the relationship between CU traits and social withdrawal*.

**Hypothesis** **3.** *Teacher–child relationship quality negatively moderates the relation between CU traits and emotion regulation, or teacher–child relationship quality constricts the effect of CU traits on this relation.*

## 2. Materials and Methods

### 2.1. Participants and Procedure

The teachers of a total of 520 young kindergarten LBC from rural areas were recruited through purposeful sampling. Eventually, data from 513 preschool LBC aged 3 to 6 years were selected and analyzed. After excluding 7 incomplete questionnaires, the response rate was 98.7%. Among these children, 257 were boys (*M*_age_ = 4.19, *SD* = 0.82) and 256 girls (*M*_age_ = 4.21, *SD* = 0.87). The proportion of LBC by age was 25.2% (age 3 and younger), 30.6% (age 4 to 5), and 44.3% (age 5 and older). The study protocol was approved by the research ethics committee of Guangzhou University (Protocol Number: GZHU202301).

### 2.2. Measures

#### 2.2.1. CU Traits

CU traits were measured using an 11-item version of the Inventory of Callous–Unemotional Traits (ICU) (Frick, 2003; Deng et al., 2016). The score of each item in two dimensions (callousness and uncaring) was reported using a 4-point Likert scale (0 = not at all true, 1 = somewhat true, 3 = very true, and 4 = definitely true). A higher score indicated a higher degree of perception of CU traits. The ICU has been examined in preschool children (Ezpeleta et al., 2013), and the revised form has shown great reliability and validity in Chinese preschool children (Zhu et al., 2023; Cao et al., 2023). Cronbach’s α of the scale in the current study was 0.79.

#### 2.2.2. Emotional Regulation

Emotion regulation was measured using the Emotion Regulation Subscale of the Emotion Regulation Checklist (ERC) (Shields & Cicchetti, 1997). There are 5 items of the revised Chinese version for preschool children (Zeng, 2020), contextually representing emotional expression, empathy, self-awareness of emotions, etc. A 4-point scale was used (0 = never, 3 = almost always). Great reliability and validity of this scale have been identified in Chinese preschool children (Zhu et al., 2020; W. Zhang et al., 2019). In this study, Cronbach’s α of this scale was 0.78.

#### 2.2.3. Social Withdrawal

Social withdrawal was measured using the Child Social Preference Scale (CSPS) (Coplan et al., 2004). The CSPS contained 11 items and divided into two dimensions: shyness (7 items) and unsociability (4 items) (Y. Li et al., 2016). This scale was scored on a 5-point Likert scale, ranging from 1 (not at all) to 5 (a lot), where the higher the total score of the CSPS, the more evident the prevalence of social withdrawal. Existing studies have examined the reliability and validity of the CSPS in Chinese preschool children (Chen & Santo, 2016; Y. Li et al., 2016; Zhu et al., 2017). In this study, Cronbach’s α of this scale was 0.89.

#### 2.2.4. Teacher–Child Relationship Quality

Teacher–child relationship quality was measured using the 15-item short form of the Student–Teacher Relationship Scale (STRS) (Pianta & Steinberg, 1992). After excluding the dependence scale with low reliability, teacher–child relationship quality was scored by the closeness and conflict scales (X. Zhang, 2011). Each item was answered using a 5-point scale ranging from 1 (definitely does not apply) to 5 (definitely applies). A higher score showed a higher quality of the relationships between preschool LBC and their teachers. Prior studies have identified the great reliability of the STRS in preschool LBC (Yang et al., 2021; Hou et al., 2019). In the current study, the internal consistency of Cronbach’s α was 0.86.

### 2.3. Statistical Analysis

In this research, the analyses of data were performed using SPSS 26.0. First, a preliminary analysis of the Pearson correlations among the measurement variables was conducted. Second, the moderated mediation effect was examined by the PROCESS macro in SPSS (Hayes, 2013). The indirect effect of emotion regulation linking CU traits and social withdrawal was determined using the macro Model 4. To estimate the standard error and confidence interval, bias-corrected bootstrapping based on 5000 samples was performed. Third, the moderated mediation effect was assessed using PROCESS (model 7) to identified whether teacher–child relationship quality weakened the effect of CU traits on emotion regulation. Meanwhile, the conditional indirect effects were tested. Gender and age were controlled for as covariates in the actual analysis.

## 3. Results

### 3.1. Preliminary Analyses

A difference test was conducted to control for the effects of age and sex. The results showed that CU traits had significant gender differences (*t*_(511)_ = 2.14, *p* < 0.05, *Cohen’s d* = 0.19), implying that the level of CU traits in left-behind boys (*M*_boys_ = 2.10, *SD* = 0.41) was significantly higher than that in girls (*M*_girs_ = 2.02, *SD* = 0.40). The quality of the teacher–child relationship also reached marginal significance in terms of gender (*t*_(511)_ = −1.93, *p* = 0.05, *Cohen’s d* = 0.17), which meant that left-behind girls (*M*_girs_ = 3.92, *SD* = 0.62) had a higher relationship quality with their teachers than boys (*M*_boys_ = 3.81, *SD* = 0.60). Regarding age, there were significant differences in CU traits (*F* = 3.26, *p* < 0.05, *η*^2^ = 0.03) and social withdrawal (*F* = 3.21, *p* < 0.05, *η*^2^ = 0.03), suggesting that increasing age among left-behind preschoolers is associated with higher risks of exhibiting CU traits and social withdrawal. In the subsequent tests, age and sex were included as covariates to examine their effects.

Further statistical correlation tests found that, as shown in Table 1, CU traits had a significant negative relationship with emotion regulation (*r* = −0.45, *p* < 0.001) and teacher–child relationship quality (*r* = −0.68, *p* < 0.001). However, there was a significant positive correlation between CU traits and social withdrawal (*r* = 0.49, *p* < 0.001). Emotion regulation was negatively correlated with social withdrawal (*r* = −0.39, *p* < 0.001) and positively correlated with teacher–child relationship quality (*r* = 0.47, *p* < 0.001). In addition, children’s social withdrawal was negatively correlated with teacher–child relationship quality (*r* = −0.59, *p* < 0.001). LBC with higher CU traits may have lower levels of teacher–child relationship quality and emotion regulation, as well as higher levels of social withdrawal behavior.

### 3.2. Testing for a Mediation Effect

By controlling for sex and age as covariates, the analysis revealed statistically significant positive effects of CU traits on social withdrawal, where *β* = 0.66, *p* < 0.001. This finding implied that CU traits could positively predict the prevalence of social withdrawal in young patients with LBC. CU traits negatively predicted emotion regulation (*β* = −0.62, *p* < 0.001), while emotion regulation negatively predicted behavioral responses to social withdrawal (*β* = −0.27, *p* < 0.001). The analysis also examined the indirect effect of emotion regulation as a mediator between CU traits and social withdrawal. The results of bias-corrected bootstrap testing revealed that the mediation effect of emotion regulation was 0.17 (*SE* = 0.04, *Boot CI* = [0.09, 0.25]), accounting for 20.1% of the total effect (*β* = 0.83, *p* < 0.001). Therefore, Hypotheses 1 and 2 were supported, suggesting that emotion regulation plays a mediating role between CU traits and social withdrawal.

### 3.3. Testing for a Moderated Mediation Effect

The moderated mediation model was further tested, and the results are shown in Table 2. CU traits were positively linked to social withdrawal (*β* = 0.66, *p* < 0.001) but negatively associated with emotion regulation (*β* = −0.31, *p* < 0.001). Conversely, teacher–child relationship quality positively predicted the development of emotion regulation (*β* = 0.30, *p* < 0.001). More importantly, the interaction effect of CU traits and teacher–child relationship quality significantly and negatively predicted the developmental outcome of emotion regulation (*β* = −0.25, *p* < 0.01). Moreover, emotional regulation was negatively related to social withdrawal (*β* = −0.27, *p* < 0.001). Therefore, teacher–child relationship quality may play a moderating role when emotion regulation acts as a mediator between CU traits and social withdrawal.

**Figure 1:**
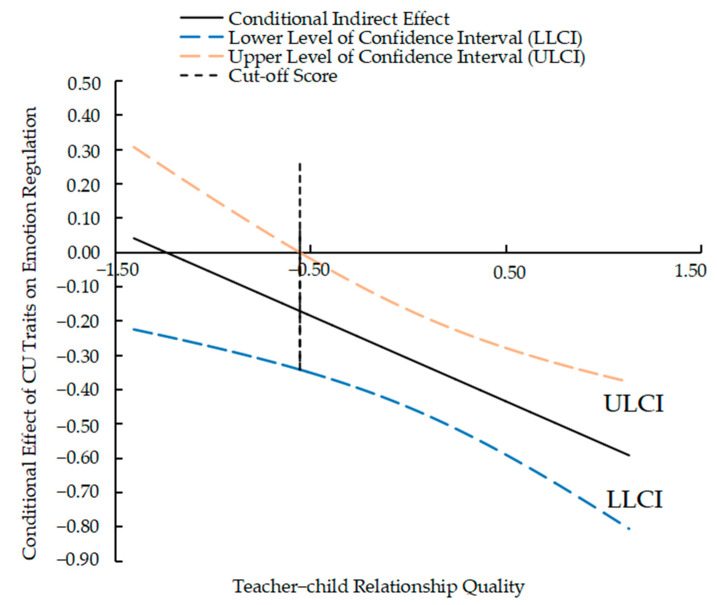
Johnson–Neyman plot of the moderated effect.

The indirect effect of emotion regulation under high, medium, and low levels of teacher–child relationship quality should be assessed. The indirect effect of emotion regulation was not significant in lower-quality teacher–child relationships (effect_lower_ = 0.04, 95% *CI* = [−0.02, 0.11]). The conditional indirect effect was significant in the average (effect_average_ = 0.09, 95% *CI* = [0.03, 0.15]) and higher-quality teacher–child relationships (effect_higher_ = 0.12, 95% *CI* = [0.06, 0.19]). Specifically, as assessed through the Johnson–Neyman technique (Figure 1), the indirect mediation effect reached statistical significance when the standard score of teacher–child relationship quality was at or above −0.5543. Therefore, an improvement in teacher–child relationship quality may reduce the effect of CU traits on emotion regulation in young LBC. A moderated mediation model involving CU traits, emotion regulation, social withdrawal, and teacher-child relationship quality was supported.

## 4. Discussion

Based on theoretical models and empirical work, this study explored a moderated mediation model in which emotion regulation could act as a mediator and bridge the path between CU traits and social withdrawal in young LBC. Additionally, the quality of the teacher–child relationship played a moderating role in weakening the adverse effects of CU traits on emotion regulation.

The early social adjustment of LBC has been a major public issue, particularly regarding the prevalence of social withdrawal in young LBC identified in a recent study (J. Zhang et al., 2018). Notably, social withdrawal is sometimes an early symptom of a number of neuropsychiatric disorders, even appearing long before other symptoms (Porcelli et al., 2019). This phenomenon may occur in the early stages of some behavioral and personality traits related to psychopathy, such as CU traits. For example, it was generally found that CU traits were important for diagnosing and predicting externalizing symptoms in children (Longman et al., 2016). Similarly, a predictive role of CU traits in children’s social functioning and peer interactions has been gradually proposed (Waller & Wagner, 2019; Haas et al., 2018; Barry et al., 2008).

The present study found that CU traits can significantly and positively predict the occurrence of social withdrawal, which is consistent with the proposals of existing theoretical models from other researchers (Facci et al., 2023; Levine et al., 2023; Wagner et al., 2020; Hawes et al., 2019; Waller & Wagner, 2019; Haas et al., 2018). For instance, according to the STAR model, children with high CU traits are characterized by a deficit in social affiliation and show low sensitivity and less desire to seek out or enjoy sustained adaptive friendships (Waller & Wagner, 2019). In other words, these children may be used to, or even enjoy, spending time alone without actively seeking meaning and value from their peers and social groups (Haas et al., 2018; Foulkes et al., 2014); peer functioning may not be conducive to their own social manipulation (Hawes et al., 2019). Consequently, for children with high CU traits, social withdrawal may be an appropriate behavioral response and reaction strategy in social situations.

For young LBC, the transformation of the family and parental migration became risk factors for affection deficits and solitary preferences (Wang et al., 2019; Van den Akker et al., 2014). Secondary CU traits are believed to result from affective deficits caused by pathogenic environmental conditions (Craig et al., 2023), especially further impairments in emotional manipulation (Bennett & Kerig, 2014). Thus, the adaptive regulation of emotions may be particularly affected. Emotion regulation mainly reflects children’s appropriate emotional expression, empathy, and emotional self-awareness in certain situations (Shields & Cicchetti, 1997). However, difficulties in emotion regulation may contribute to delays in developing adaptive behaviors, which, in turn, may inhibit social interactions with peers (Calkins et al., 1999; Raver et al., 1999).

According to the findings of this study, emotion regulation as a proximate variable mediated the effect of CU traits on social withdrawal. This is consistent with existing theoretical views (Shao & Zhang, 2021; Squillaci & Benoit, 2021; Craig & Moretti, 2019). Notably, exposed to adverse parenting environments, children with high CU traits may develop a “cold” mask as an approach to coping with social situations (Craig et al., 2023), likely with inflexible and negative emotional expression and feedback (Craig & Moretti, 2019). In other words, the emotional regulation of young LBC could act as an intermediate process between personal factors (e.g., CU traits) and the withdrawn response. Due to insecure attachment from absent parents, LBC might not be very keen on the adaptive response and regulation of social emotions (Qu et al., 2020). Consequently, they are reluctant to participate in social interactions with their peers and show withdrawn and solitary responses.

Although there might be a recursive relationship between attachment and CU traits in the process of social adjustment (Baroncelli et al., 2024), a compensatory effect occurs in early growing environments, such as kindergartens and families (Y. F. Li, 2022). However, it is believed that a high-quality kindergarten environment will reduce the impact of negative family environments and safeguard the positive development of disadvantaged children. The bioecological model also provides a basis for understanding such compensatory effects between growth environments (Bronfenbrenner & Morris, 2006), and a high quality of the teacher–child relationship is considered an important source of the compensating effect (Y. F. Li, 2022).

The moderated mediation model in this study verified that a high-quality teacher–child relationship could weaken the negative effect of CU traits on emotional and social adjustment, which provided new validation of the compensatory effect from the perspective of psychopathology and early developmental environments. The teacher–child relationship was regarded as the potential point of effective intervention in addressing CU traits (Baroncelli & Ciucci, 2020; Barry et al., 2008). For example, teachers can form high-quality social relationships with children with high CU traits (J. L. Allen et al., 2018). More specifically, high-quality teacher–child relationships can improve the resilience of young LBC with parental absence and further reduce the prevalence of social withdrawal (Liu et al., 2015; Yang et al., 2021). Therefore, more attention should be paid to the unique significance of the teacher–child relationship in the socialization and peer functioning of LBC.

Left-behind preschool children are expected to achieve the appropriate developmental processes and outcomes of early socialization. Future studies should explore the association between CU traits and the growth environment of LBC in early childhood. In particular, it is clear that future longitudinal developmental studies will contribute more valuable evidence for providing more emotional support to left-behind preschool children with CU traits.

## 5. Conclusions

Although CU traits are known to be significant predictors of conduct disorders in mid–late childhood and adulthood, low prosociality and withdrawn responses in early developmental stages may also be the first signs in children suffering from parental migration. The current study explored the social withdrawal of left-behind preschool children from a comprehensive perspective of psychopathology and early growth environments. Not only did CU traits predict social withdrawal, but emotion regulation and teacher–child relationships also played important indirect roles in the model. Based on the findings of this study, future research can adopt a longitudinal design to explore how CU traits and social withdrawal interact and evolve over time. For left-behind young children, there is still more room to explore the significant role of high-quality teacher–child relationship and CU traits on their emotional and social development.

## Figures and Tables

**Table 1:** Pearson two-tailed correlations for all variables.

Variables	*M*	*SD*	1	2	3	4	5	6
1. CU traits	2.06	0.41	—					
2. Emotion regulation	2.66	0.57	0.45 ***	—				
3. Social withdrawal	2.10	0.69	0.49 ***	0.39 ***	—			
4. Teacher–child relationship quality	3.87	0.61	0.68 ***	0.47 ***	0.59 ***	—		
5. Gender	0.50	0.50	0.09 *	−0.05	−0.01	−0.09	—	
6. Age	4.20	0.84	−0.08	0.08	−0.04	0.004	0.10	—

N = 513. Gender is a dummy variable: boy = 1, girl = 0. * *p* < 0.05. *** *p* < 0.001.

**Table 2:** Testing the moderated mediation effects.

	Emotion Regulation				Social Withdrawal			
	*β*	*SE*	*t*	95% *CI*	*β*	*SE*	*t*	95% *CI*
CU traits	−0.31	0.07	−4.28 ***	[−0.45, −0.16]	0.66	0.07	9.25 ***	[0.37, 0.52]
TCRQ	0.30	0.05	6.25 ***	[0.21, 0.40]				
CU traits × TCRQ	−0.25	0.08	−3.22 **	[−0.40, −0.10]				
Emotion regulation					−0.27	0.05	−5.18 ***	[−0.37, −0.17]
Gender	0.004	0.04	0.91	[−0.08, 0.09]	−0.08	0.05	−1.46	[−0.18, 0.03]
Age	0.04	0.02	0.16	[−0.01, 0.09]	0.01	0.03	0.33	[−0.05, 0.07]
*R* ^2^	0.27				0.28			
*F*	38.35 ***				48.82 ***			

N = 513. TCRQ indicates teacher–child relationship quality. ** *p* < 0.01. *** *p* < 0.001.

## Data Availability

The original contributions presented in this study are included in the article. Further inquiries can be directed to the corresponding author.
